# Usefulness of computed tomography–guided puncture biopsy coupled with rapid on‑site evaluation for diagnosis of pulmonary lesions: a systematic review and meta‑analysis

**DOI:** 10.20452/wiitm.2024.17895

**Published:** 2024-07-31

**Authors:** Zhongbao Zhang, Rui Liu, JunLin Li, Kai Zhang, Yuan Li, Xiaoqin Zhang, Sanjay Rastogi

**Affiliations:** Department of Imaging Medicine, People’s Hospital of Inner Mongolia Autonomous Region, Inner Mongolia, Hohho, China; Boston University Medical Campus, Boston, Massachusetts, United States

**Keywords:** computed tomography–guided, lung biopsy, rapid on‑site evaluation, systematic evaluation

## Abstract

**INTRODUCTION:**

Accurate identification of lung lesions during lung biopsy (LB) surgery can be achieved with the use of computed tomography (CT) guidance. The rapid on‑site evaluation (ROSE) method allows for quick assessment of the features, cytomorphological traits, and appropriateness of the obtained tissue samples, and might further accelerate the diagnostic workup.

**AIM:**

We aimed to investigate the diagnostic value of CT‑guided aspiration biopsy combined with ROSE for assessment of pulmonary lesions.

**MATERIALS AND METHODS:**

A PubMed and Embase search was undertaken until October 2023 to find studies on lung lesion diagnosis utilizing CT‑guided needle biopsy and ROSE. The main method for assessing bias and relevance was the updated Quality Assessment of Diagnostic Accuracy Research 2 tool. The threshold effect and subgroup analysis were used to determine the source or heterogeneity. Sensitivity, specificity, diagnostic odds ratio (DOR), area under the summary receiver operating characteristics curve (SROC AUC), and the Q‑index were calculated. The Deek funnel plot was used to evaluate publication bias.

**RESULTS:**

A total of 6 studies (n = 951) with mild heterogeneity were included in this meta‑analysis, yielding a pooled sensitivity, specificity, and DOR of 0.94 (95% CI, 0.91–0.96), 0.95 (95% CI, 0.9–0.98), and 159.05 (95% CI, 69.59–363.49), respectively. The SROC AUC, calculated using a random‑effects model, was 0.98. Subgroup analysis showed that study design (prospective vs retrospective) had an impact on sensitivity. Further analysis of 3 studies that established control groups showed that the ROSE group had by 12% (95% CI, 0.08–0.16; I^2^ = 0) higher sampling adequacy and diagnostic accuracy than the non‑ROSE group, while there was no significant difference in the rate of complications.

**CONCLUSIONS:**

For assessment of pulmonary lesions, CT‑guided puncture biopsy combined with ROSE has high sensitivity, specificity, and diagnostic accuracy, and is a practical operational method that merits wide clinical application.

## INTRODUCTION 

Lung cancer is a common malignancy and a major cause of cancer‑related death worldwide.^1^ Due to widespread use of low‑dose computed tomography (CT), the number of detected lung lesions has significantly increased over the past few years. The overlapping imaging features of old and new lesions make it challenging to differentiate benign lesions from malignantones, emphasizing the importance of assessing the lesion nature, which could impact patient prognosis and quality of life.

A time‑honored technique for reliable diagnosis of lung nodules and malignancies is lung biopsy (LB).^2^^;^^3^^;^^4^ Its diagnostic accuracy ranges from 65% to 94%, depending on the study.^5^^;^^6^^;^^7^^;^^8^These diagnostic yields are affected by a number of variables, including the size of the lesion, the type of needle used (core vs small needle), and the imaging guiding approach (bronchoscopy, CT, or CT fluoroscopy).^6^^;^^7^^;^^8^^;^^9^ It has also been claimed that insufficient biopsy material can be the cause of lung cancer misdiagnosis.^10^^;^^11^

CT‑guided percutaneous transthoracic needle biopsy (PTNB) was first described in 1976. The procedure can be divided into 2 types: fine needle aspiration (FNA) and core needle biopsy (CNB), depending on the biopsy needle used. PTNB is increasingly recognized as a minimally invasive method for diagnosing lung lesions, especially peripheral ones. However, its limitations include inadequate sampling of some lesions and the risk of false negative results.^12^ Accordingly, it is essential to identify a new approach to determine whether the lesion tissue is successfully punctured.

The rapid on‑site evaluation (ROSE) technique was proposed in 1981 as a method that would provide immediate feedback on the adequacy of sample acquisition during the examination, guide the operator in modifying the sampling technique (such as the site and depth of sampling), and allow for rapid diagnosis. However, no consensus has been reached on whether ROSE can improve diagnostic accuracy. In this respect, Liu et al^13^ reported that ROSE did not improve the pathological diagnosis rate of the endobronchial ultrasound (EBUS)‑guided transbronchial needle aspiration (TBNA) procedure. Monaco et al^14^ concluded that ROSE did not affect the diagnostic rate of EBUS‑FNA but ensured validity and adequacy of sampling, providing more adequate specimens for subsequent tests, such as flow cytometry, immunostaining, and molecular pathology.

ROSE is a useful tool for swift evaluation of the cytomorphologic characteristics of biopsy specimens for sufficiency and malignancy. To improve the precision of LB diagnosis, ROSE procedures should be carried out under the supervision of experienced pathologists.^11^ Biopsies guided by bronchoscopy frequently make use of ROSE techniques.^11^^;^^15^^;^^16^^;^^17^^;^^18^^;^^19^ On the other hand, research on their application in CT‑guided LB has been sparse.^20^^;^^21^^;^^22^^;^^23^^;^^24^^;^^25^^;^^26^

By taking into account different types of bias that might impact individual results, meta‑analyses help decrease the risk of bias and increase the statistical power of the results.^27^ Little is currently known on whether CT‑guided puncture biopsy combined with ROSE has guiding significance for diagnosing pulmonary lesions. Therefore, this study reviewed the medical literature to evaluate the diagnostic value of CT‑guided puncture biopsy combined with ROSE for assessment of pulmonary lesions. Moreover, we sought to explore the complications of this procedure to provide a basis for the selection of a clinically optimal diagnostic approach.

## AIM 

The purpose of this study was to investigate the safety and diagnostic accuracy of using a combination of CT‑guided LB and ROSE techniques for assessing lung lesions.

## MATERIALS AND METHODS 

This systematic review and meta‑analysis adheres to the reporting guidelines outlined in the Preferred Reporting Items for Systematic Reviews and Meta‑analysis (PRISMA) statement.^28^

### Inclusion criteria 

The inclusion criteria were related to 4 domains: 1) study type—studies published in English that evaluated the diagnostic value of CT‑guided LB combined with ROSE for the assessment of pulmonary lesions; 2) study population—patients with known lung lesions prior to CT‑guided puncture biopsy; 3) diagnostic criteria—given that ROSE involves rapid staining of cell smear and does not allow for lesion morphology assessment, it cannot effectively distinguish the pathologic type of lung cancer, and can only offer preliminarily information on whether the tissue is benign or malignant. Therefore, we only analyzed studies in which the final results of CT‑guided puncture biopsy combined with ROSE were compared with the gold standard, that is, surgical and histopathologic findings. 4) evaluation index—sufficient data could be found in or calculated from the original study, such as true positive rate, true negative rate, false positive rate, false negative rate, the rate and type of complications, and the adequacy of sampling.

### Exclusion criteria

Studies were not eligible for analysis if the diagnosis was not confirmed by the abovementioned gold standard. Review articles, letters, animal studies, and case reports were also excluded.

### Search strategy

We searched the PubMed and Embase databases from inception until October 2023 using the following key words and related medical subject heading terms: *Biopsy, Needle AND Tomography, X‑Ray Computed AND Rapid On‑site Evaluation AND Lung Neoplasms*. Only English‑language publications were considered. We also accessed additional published, unpublished, and investigational studies via the following methods: 1) the “Related articles” function of PubMed was used to identify potentially relevant publications linked to the retrieved studies; 2) the Science Citation Index was searched to obtain papers cited in the retrieved studies; 3) reference lists of the included studies were manually searched.

### Data extraction and quality assessment

Firstly, 2 authors (JL and KZ) independently reviewed the titles and abstracts of the retrieved publications to identify potentially relevant articles. Data were extracted using a predesigned form, and any disagreements were resolved through discussion. The following data were extracted: 1) basic characteristics of included studies: first author, date of publication, title, journal name, country; 2) study population, design, sample size, diagnostic method, and rapid staining method; and 3) evaluation index: 4‑grid table data (true‑positive, false‑positive, true‑negative, and false‑negative rates).

Quality assessment was performed using the updated Quality Assessment of Diagnostic Accuracy Research (QUADAS‑2) tool,^29^ and each study was evaluated for the risk of bias by assigning an answer “yes,” “no,” or “unclear” to the predefined signaling questions. The risk of bias was determined as low, high, or uncertain.

### Statistical analysis 

During meta‑analysis of diagnostic tests, the threshold effect plays an important role in determining the heterogeneity of accuracy. The threshold effect was calculated using the Spearman rank correlation coefficient between the sensitivity (true‑positive rate) and specificity. When the threshold effect was absent, heterogeneity was further analyzed using the χ^2^ test, and the magnitude of heterogeneity was quantified by I^2^. If I^2^ was lower than 50%, a fixed‑effects model was used for the combined analysis. Otherwise, a random‑effects model was used, and the source of heterogeneity was determined by subgroup analysis. Finally, sensitivity, specificity, diagnostic odds ratio (DOR), area under the summary receiver operating characteristics curve (SROC), and the Q‑index were calculated. A greater Q‑index indicated higher accuracy of the diagnostic test. The Deek funnel plot was used to evaluate publication bias. All statistics in this systematic evaluation were analyzed using Stata13 (StataCorp, College Station, Texas, United States), RevMan 5.3 (The R Foundation for Statistical Computing, Vienna, Austria), and Meta‑disc 1.4 (Metadisc, Madrid, Spain) software. A P value below 0.05 was considered significant.

## RESULTS

### Screening and inclusion of literature

**Figure 1:**
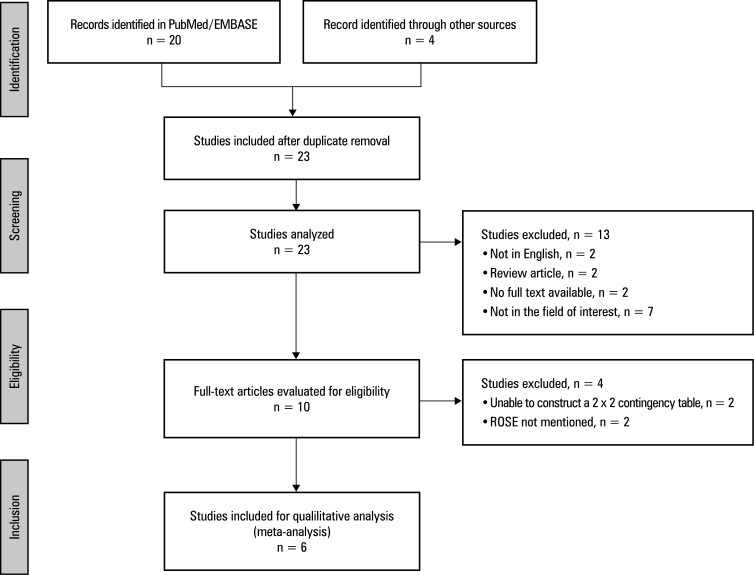
Study search and selection flowchart

A total of 19 studies were retrieved from the PubMed and Embase databases, and additional 4 were identified via manual searching of reference lists of the retrieved papers. Eleven studies were excluded based on irrelevant title and abstract, and another 6 were rejected after full‑text evaluation. Finally, 6 articles ^24^^;^^30^^;^^31^^;^^32^^;^^33^^;^^34^ met the criteria for inclusion in our meta‑analysis. The flow chart of the literature screening process is shown in (Figure 1).

### Summary of study characteristics

The 6 studies included 1179 patients, and histopathologic results were available for 951 individuals (80.7%). In the study by Fassina et al,^31^ 225 out of 311 participants did not undergo a histopathologic examination, whereas in the study by Santambrogio et al,^34^ 3 patients underwent radiologic follow‑up 15 to 21 months after biopsy, and were finally included in the true‑negative group since no changes in the lesions were observed. Four studies were prospective,^24^^;^^32^^;^^33^^;^^34^ 2 were retrospective,^30^^;^^31^ and 4 included a control group.^24^^;^^32^^;^^33^^;^^34^

**Figure 2:**
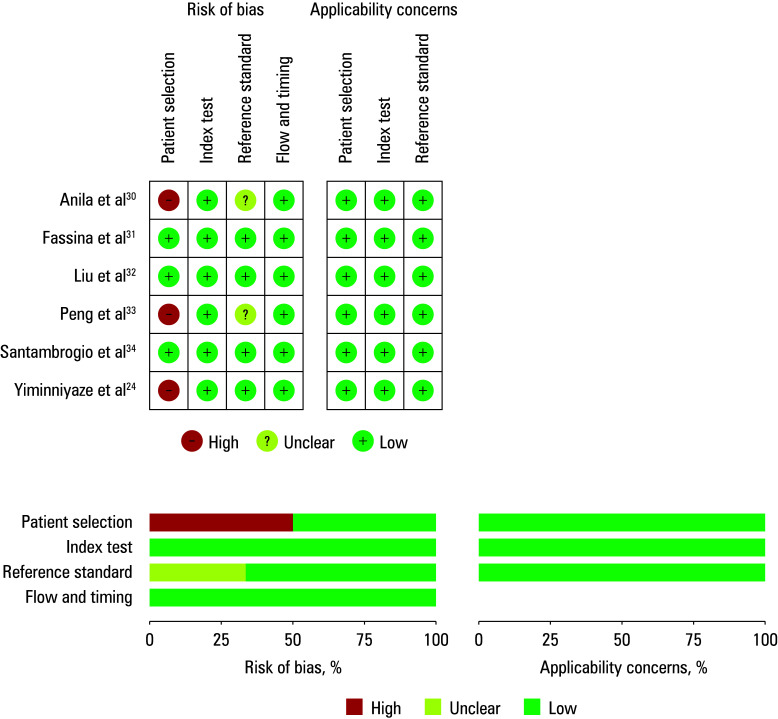
Reporting quality assessment by the Quality Assessment of Diagnostic Accuracy Research‑2 scoring system

The sampling methods encompassed FNA in 5 studies^30^^;^^31^^;^^32^^;^^33^^;^^34^ and CNB in 1 paper.^24^ Diverse reagents were used for ROSE staining, including the Diff reagent,^24^^;^^32^^;^^33^ toluidine blue reagent,^30^ methylene blue reagent,^11^ and the Kimsa reagent.^34^ ROSE was performed by a cytopathologist in all studies. Raw data are summarized in (TABLE 1  and TABLE 2 ). Methodological quality evaluation of the included studies is shown in (Figure 2).

**TABLE 1:** Characteristics of the included studies

Study	Country	Study design	Sample size, n	Biopsies performed, n	Main sampling site	ROSE reagent	ROSE reporter	Sampling method
Anila et al^30^	India	PCS	50	50	Masses	Toluidine blue	Pathologist	FNA
Fassina et al^31^	Italy	PCS	311	86	–	Giemsa	Pathologist	FNA
Liu et al^32^	China	RCT	108	108	Nodules	Diff‑quik	Pathologist	FNA
Peng et al^33^	China	RCS	205	205	Nodules / masses	Diff‑quik	Pathologist	FNA
Santambrogio et al^34^	Italy	RCT	220	207	Nodules	Giemsa	Pathologist	FNA
Yiminniyaze et al^24^	China	RCS	285	285	Nodules / masses	Diff‑quik	Pathologist	CNB

Abbreviations: CNB, core needle biopsy; FNA, fine-needle aspiration; PCS, prospective cohort study; RCS, retrospective cohort study; RCT, randomized controlled trial; ROSE, rapid on‑site evaluation

**TABLE 2:** Accuracy of rapid on‑site evaluation for diagnosis of pulmonary lesions

Study	Patients, n		Adequacy		Complications		TP	FP	FN	TN
	ROSE	Non‑ROSE	ROSE	Non‑ROSE	ROSE	Non‑ROSE				
Anila et al^30^	50	–	39	–	Pneumothorax (n = 3)	–	31	0	3	16
Fassina et al^31^	311	–	305	–	Pneumothorax (n = 13), hemoptysis (n = 4), chest pain (n = 3)	–	77	0	3	6
Liu et al^32^	56	52	52	41	Pneumothorax (n = 6), hemoptysis (n = 10)	Pneumothorax (n = 7), hemoptysis (n = 11)	28	2	4	22
Peng et al^33^	132	102	–	–	Pneumothorax (n = 9), hemoptysis (n = 2)	Pneumothorax (n = 15), hemoptysis (n = 2)	57	4	7	64
Santambrogio et al^34^	110	110	110	97	Pneumothorax (n = 29)	Pneumothorax (n = 23)	63	1	7	26
Yiminniyaze et al^24^	163	122	160	105	Pneumothorax (n =34), hemoptysis (n = 21)	Pneumothorax (n = 16), hemoptysis (n = 11)	150	0	3	6

Abbreviations: FN, false-negative rate; FP, false-positive rate; TN, true-negative rate; TP, true-positive rate; others, see TABLE 1

### Threshold effect

The SROC curve did not exhibit a “shoulder‑arm” distribution, and the Spearman correlation coefficient between log sensitivity and log 1‑specificity was 0.83 (*P* >0.05), indicating no threshold effect in this meta‑analysis.

### Meta-analysis results

**Figure 3:**
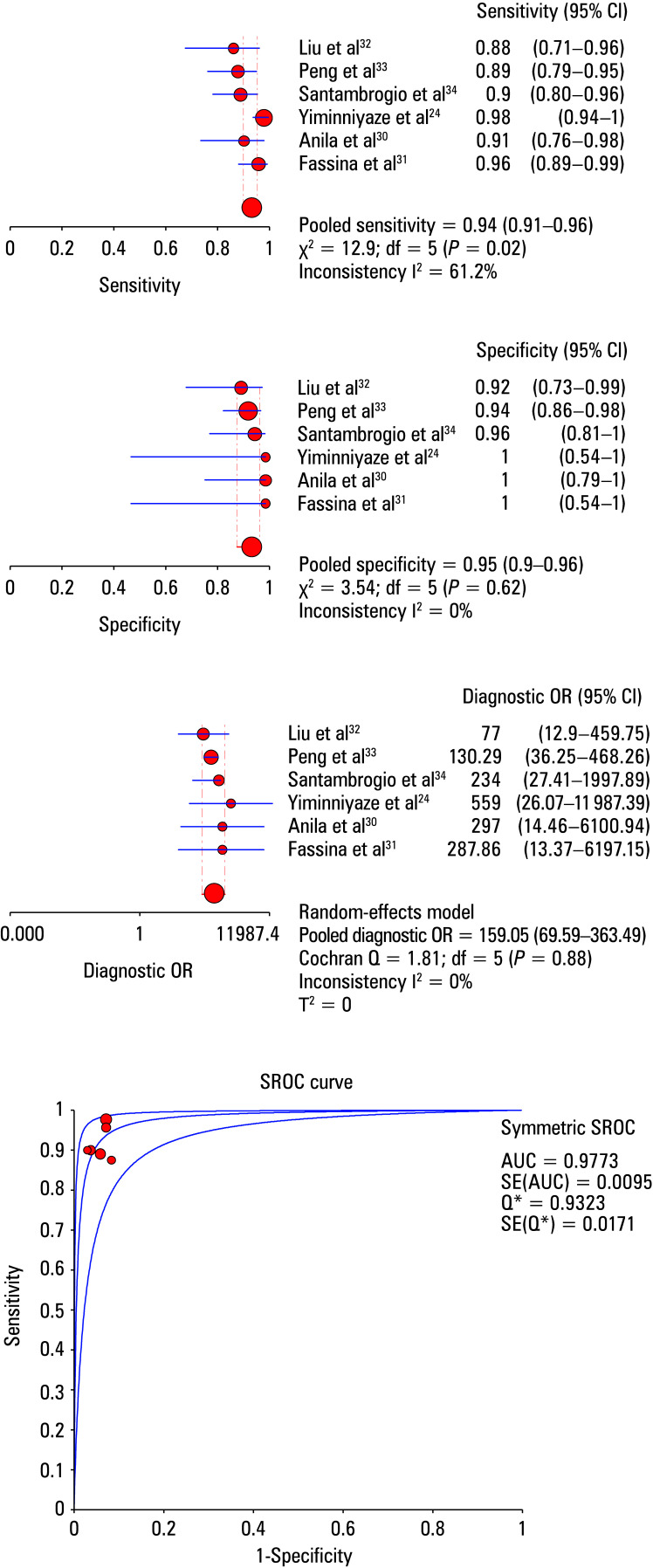
Forest plot showing the sensitivity, specificity, diagnostic odds ratio, and summary receiver operating characteristics (SROC) analysis of the 6 included studies and the pooled estimates

Abbreviations: AUC, area under the curve; df, degree of freedom; OR, odds ratio

The heterogeneity test showed mild heterogeneity among the studies for sensitivity (χ^2^ = 12.9; I^2^ = 61.2%; *P* = 0.02), and the effect sizes were calculated using a random‑effects model. No heterogeneity was observed for specificity (χ^2^ = 3.54; I*^2^* = 0; *P *= 0.62) and DOR (χ^2^ = 1.81; I^2^ = 0; *P* = 0.88), thus, a fixed‑effects model was applied. The pooled sensitivity, specificity, and DOR were 0.94 (95% CI, 0.91–0.96), 0.95 (95% CI, 0.9–0.98), and 159.05 (95% CI, 69.59–363.49), respectively. The SROC AUC was 0.98, and the Q‑index was 0.93 (Figure 3).

### Subgroup analysis

**Figure 4:**
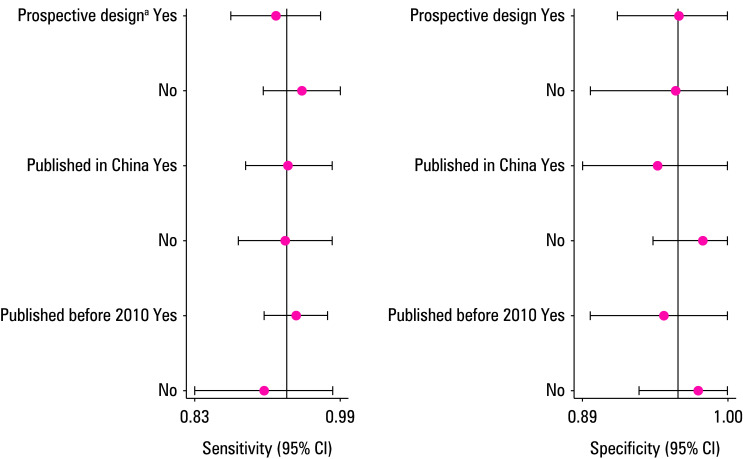
Univariable meta-regression and subgroup analyses for sensitivity

Subgroup analysis was conducted based on the study type (prospective vs retrospective), country of publication (China vs non‑China), and year of publication (before vs after 2010). Heterogeneity of sensitivity among the studies was related to the study type, and sensitivity of retrospective studies was significantly higher than that of prospective ones. More details are shown in (Figure 4).

a *P* <0.05

### Adequacy of sampling, diagnostic accuracy, and complications

**Figure 5:**
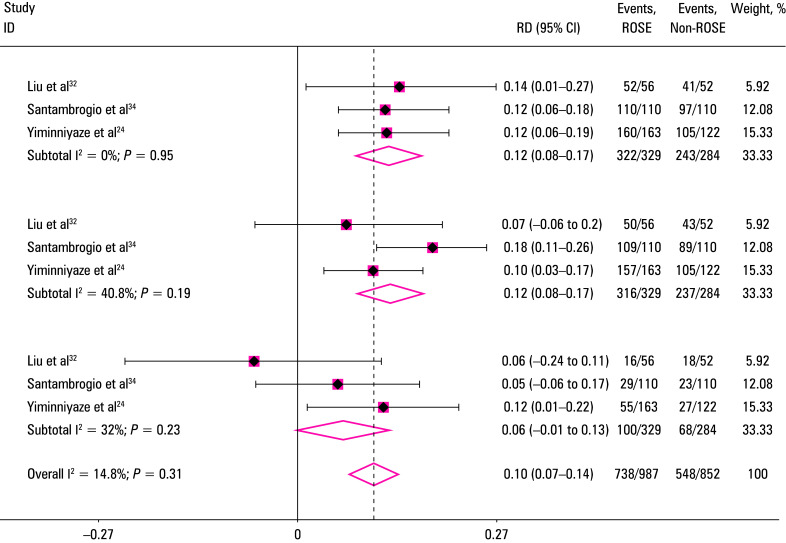
5 Forest plots comparing the adequacy rate (**A**), diagnostic accuracy (**B**), and the incidence of complications (**C**) with or without rapid on‑site evaluation in the included studies

Abbreviations: RD, risk difference; others, seeTABLE 1

A total of 4 studies^24^^;^^32^^;^^33^^;^^34^ established control groups, but 1 of them^33^ did not specify sampling adequacy and diagnostic accuracy.Figure 5 shows the results of sampling adequacy and diagnostic accuracy analysis in the ROSE vs non‑ROSE groups in the 3 relevant studies. Application of ROSE resulted a 12% improvement in sampling adequacy (95% CI, 0.08–0.16; I^2^ = 0), while the diagnostic accuracy increased by 13% (95% CI, 0.06–0.19; I^2^ = 41%). The incidence of complications was similar between the ROSE and non‑ROSE groups.

### Publication bias

**Figure 6:**
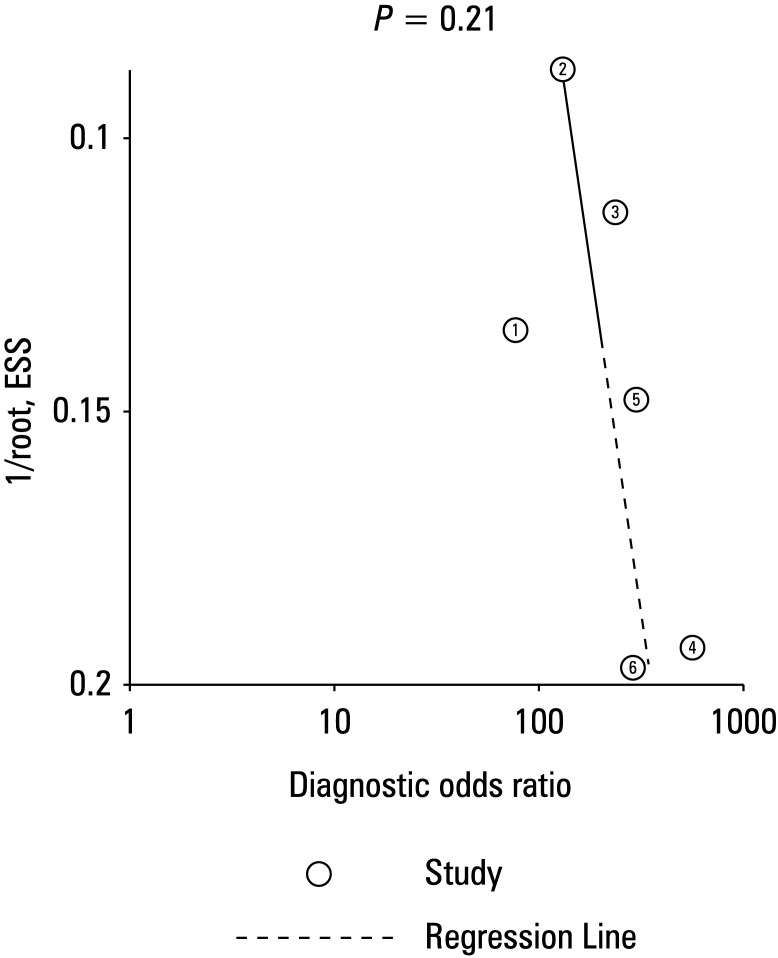
The Deek funnel plot asymmetry test for publication bias of the 6 studies included in the meta‑analysis

Abbreviations: ESS, effective sample size

Deek funnel plots were generated using a *P* value greater than 0.1 to indicate significant publication bias. No publication bias was observed, as shown in (Figure 6).

## DISCUSSION

The present study sought to assess the diagnostic value of ROSE in lung puncture biopsy. Comparing CT‑guided lung lesion biopsies performed with and without the ROSE technique, this meta‑analysis looked at the diagnostic efficacy and safety results. Overall, we demonstrated that the use of ROSE significantly improved the accuracy of CT‑guided LB diagnosis without increasing treatment time or the rate of procedure‑related complications.

Studies analyzing various CT‑guided LB techniques have shown that improved diagnostic accuracy is the main outcome.^3^^;^^25^^;^^35^ The present study found that concurrent application of ROSE significantly improved diagnosis rates, increasing them by about 10.8 percentage points. Furthermore, it was shown that ROSE significantly decreased the incidence of secondary LB in the study patients. Early diagnoses based on ROSE can guide subsequent patient care, which is in line with ROSE’s capacity to provide fast feedback on the cytomorphological sufficiency and other features of LB samples.^32^

As compared with the definitive pathology diagnosis, the ROSE‑based diagnoses are quite consistent. The reported accuracy rates range between 89.3% and 95.7%.^24^^;^^32^ However, despite its utility in rapid cell smear staining, ROSE falls short as a definitive diagnostic tool due to its inability to differentiate between various forms of lung cancer and to provide histologic or morphologic information. It only allows clinicians to determine whether a lung lesion is malignant or benign.^32^

Clinically, CT‑guided puncture biopsy remains the mainstay for sampling lung lesions, as it is associated with low invasiveness and an acceptable complication rate. However, in some cases, the number of adequate samples is limited due to operator proficiency or lesion location and size, ^26^ resulting in missed diagnoses and delays in effective treatment.

ROSE represents a rapid cytological interpretation technique for diagnostic interventional pulmonology that saves operative time and resources, and reduces pain and complications. In the relevant 3 studies that included a control group, satisfactory specimens were obtained in 97.9% of the patients in the ROSE group (322/329), yielding an accurate diagnosis in 316 cases. In contrast, 85.6% of the patients in the non‑ROSE group (243/284) had satisfactory specimens obtained, and an accurate diagnosis was made in 237 cases. Overall, ROSE increased the adequacy of sampling and diagnostic accuracy of lung lesions by 12% and 13%, respectively, suggesting that application of this technique can improve the positivity rate and diagnostic accuracy of lung puncture, prevent further trauma, and reduce costs.

Among the 6 studies that met the inclusion criteria, the heterogeneity of ROSE’s specificity in diagnosing lung lesions was not significant, although mild heterogeneity in sensitivity was observed. Subgroup analysis showed that the heterogeneity was related to the study design. Indeed, it is well‑established that retrospective studies are associated with selection bias and time bias, both of which affect sensitivity to a certain extent. Accordingly, a random‑effects model was applied for analysis. The pooled sensitivity, specificity, and AUC were 94%, 95%, and 0.97, indicating that CT‑guided puncture biopsy combined with ROSE has a high diagnostic value for differentiating benign lung lesions from malignant ones.

The ROSE method does take time to complete the required dying and associated investigations, although this time can be significantly reduced depending on the operator’s expertise. Therefore, when the ROSE technique was integrated into the LB workflow, there were no discernible changes in the procedure length. However, this end point showed significant heterogeneity. Due to differences in experience and knowledge among operators and the wide range of possible features, retrospective analyses may be skewed. This result has to be confirmed by further well‑planned prospective trials.

Current evidence suggests that the most common complications of percutaneous puncture biopsy of chest tumors are pneumothorax, hemorrhage, and pleural reaction. Other complications, including air embolism, pericardial tamponade, and tumor needle tract implantation, are relatively rare. A small pneumothorax and light bleeding are self‑limiting and do not require special treatment. The pleural reaction may be related to sex, age, body type, emotional stress, basal glucose level, a history of transthoracic punctures, or lesion and puncture locations. In most cases, patients experience mild symptoms that resolve spontaneously without treatment.

In a large study by Yarmus et al,^36^ the incidence of pneumothorax and hemoptysis was 51.8% and 10.6%, respectively. The complications reported in the 6 studies included in our meta‑analysis comprised pneumothorax (n = 95), hemoptysis (n = 37), and chest pain (n = 3). No other complications occurred, and the incidence of adverse events was within acceptable limits (4%–26% for pneumothorax, 1.5%–17.9% for hemoptysis, and 0.96% for chest pain), with no serious consequences in any of the patients after initiation of supportive treatment. This finding emphasizes the high safety profile of CT‑guided puncture biopsy combined with ROSE for the diagnosis of pulmonary lesions.

Core needles, as opposed to tiny needles, are associated with higher levels of sample adequacy.^7^ The impact of ROSE on the diagnostic accuracy of CT‑guided CNB techniques was examined by subgroup analysis. According to the results, ROSE markedly enhanced the accuracy of these procedures, without compromising safety in any way.

### Limitations

This study followed the recommended reporting norms for a meta‑analysis of diagnostic tests. Although a meticulous literature search and data extraction were conducted, certain limitations should be acknowledged. First, a small number of studies on the application of ROSE in CT‑guided percutaneous LB was conducted in China and abroad, and these studies yielded inconsistent findings. Besides, this study did not directly compare the results of CT‑guided percutaneous puncture lung biopsy with other imaging guidance modalities. Future studies with larger sample sizes are warranted to improve the robustness of our findings.

## CONCLUSIONS 

In summary, the use of CT‑guided puncture biopsy in conjunction with prompt on‑site assessment represents a safe and practical supplementary diagnostic approach that exhibits notable levels of diagnostic precision, sensitivity, and specificity in the identification of lung lesions. However, it is important to note that the studies incorporated in this review were subject to potential bias; therefore, the findings should be interpreted with caution.

## References

[BIBR-1] Sung H., Ferlay J., Siegel R.L. (2021). Global Cancer Statistics 2020: GLOB‐ OCAN estimates of incidence and mortality worldwide for 36 cancers in 185 countries. CA Cancer J Clin.

[BIBR-2] Bourgouin P.P., Rodriguez K.J., Fintelmann F.J. (2021). Image‐guided percutaneous lung needle biopsy: how we do it. Tech Vasc Interv Radiol.

[BIBR-3] Brioulet J., David A., Sagan C. (2020). Percutaneous CT‐guided lung biopsy for the diagnosis of persistent pulmonary consolidation. Diagn Interv Imaging.

[BIBR-4] Wang S., Gao X., Hui H. (2023). Comparison between preoperative hook‐wire and liquid material localization for pulmonary nodules: a meta‐analysis. Wideochir Inne Tech Maloinwazyjne.

[BIBR-5] Fu Y.F., Li G.C., Xu Q.S. (2020). Computed tomography‐guided lung biopsy: a randomized controlled trial of low‐dose versus standard‐dose protocol. Eur Radiol.

[BIBR-6] Liu D., Zhang R., Yu X. (2023). Comparison of two methods for CT‐guided pulmonary nodule location before thoracoscopic surgery. Wideochir Inne Tech Maloinwazyjne.

[BIBR-7] Li Y., Yang F., Huang Y.Y., Cao W. (2022). Comparison between computed tomography‐guided core and fine needle lung biopsy: a meta‐analysis. Medicine (Baltimore.

[BIBR-8] Fu Y.F., Li G.C., Cao W. (2020). Computed tomography fluoroscopy‐guided versus conventional computed tomography‐guided lung biopsy: a systematic review and meta‐analysis. J Comput Assist Tomogr.

[BIBR-9] Yeow K.M., Tsay P.K., Cheung Y.C. (2003). Factors affecting diagnostic accu‐ racy of CT‐guided coaxial cutting needle lung biopsy: retrospective analysis of 631 procedures. J Vasc Interv Radiol.

[BIBR-10] Hiraki T., Mimura H., Gobara H. (2009). CT fluoroscopy‐guided biopsy of 1000 pulmonary lesions performed with 20‐gauge coaxial cutting needles: diagnostic yield and risk factors for diagnostic failure. Chest.

[BIBR-11] Huang Z., Zhuang D., Feng A. (2021). Real‐time and accuracy of rapid on‐site cytological evaluation of lung cancer. Transl Cancer Res.

[BIBR-12] Chung C., Kim Y., Park D. (2020). Transthoracic needle biopsy: how to maximize diagnostic accuracy and minimize complications. Tuberc Respir Dis (Seoul.

[BIBR-13] Liu Q.H., Arias S., Wang K.P. (2016). International association for the study of lung cancer map, Wang lymph node map and rapid on‐site evaluation in transbronchial needle aspiration. J Thorac Dis.

[BIBR-14] Monaco S.E., Pantanowitz L., Khalbuss W.E. (2012). Comparing endobronchial ultrasound‐guided fine needle aspiration specimens with and without rapid on‐site evaluation. Cytojournal.

[BIBR-15] Lin C.K., Jan I.S., Yu K.L. (2020). Rapid on‐sitecytologic evaluation by pulmonologist improved diagnostic accuracy of endobronchial ultrasound‐guided transbronchial biopsy. J Formos Med Assoc.

[BIBR-16] Chen C.H., Cheng W.C., Wu B.R. (2015). Improved diagnostic yield of bronchoscopy in peripheral pulmonary lesions: combination of radial probe endobronchial ultrasound and rapid on‐site evaluation. J Thorac Dis.

[BIBR-17] Jain D., Allen T.C., Aisner D.L. (2018). Rapid on‐site evaluation of endobronchial ultrasound‐guided transbronchial needle aspirations for the diagnosis of lung cancer: a perspective from members of the pulmonary pathology society. Arch Pathol Lab Med.

[BIBR-18] Madan K., Dhungana A., Mohan A. (2017). Conventional transbronchial needle aspiration versus endobronchial ultrasound‐guided transbronchial needle aspiration, with or without rapid on‐site evaluation, for the diagno‐ sis of sarcoidosis: a randomized controlled trial. J Bronchology Interv Pulm‐ onol.

[BIBR-19] Sehgal I.S., Dhooria S., Aggarwal A.N., Agarwal R. (2018). Impact of rapid on‐site cytological evaluation (ROSE) on the diagnostic yield of transbronchial needle aspiration during mediastinal lymph node sampling: systematic review and meta‐analysis. Chest.

[BIBR-20] Huang Y., Xia L., Tang C. (2020). Application research of cell rapid on‐site evaluation in CT‐guided percutaneous lung biopsy. Chong Qing Yi Xue.

[BIBR-21] Li Y., Li K., Wang X. (2020). Rapid on‐site evaluation in CT‐guided percutaneous biopsy of peripheral pulmonary nodules. Chin J Interv Imaging.

[BIBR-22] Wang C., Wang Y., Yang Q. (2021). The application of rapid on‐site cytological evaluation for percutaneous lung biopsy. Tianjin Med J.

[BIBR-23] Zhang L., Tao L., Wang X., Li X. (2021). Application of C‐ROSE combined with 4D navigation‐guided CT localization in percutaneous puncture lung biopsy. China Med Equip.

[BIBR-24] Yiminniyaze R., Zhang X., Zhang Y. (2022). Diagnostic efficiency and safety of rapid on‐site evaluation combined with CT‐guided transthoracic core needle biopsy in suspected lung cancer patients. Cytopathology.

[BIBR-25] Huang Y.Y., Cheng H., Li G.C. (2021). Computed tomography‐guided core needle biopsy for lung nodules: low‐dose versus standard‐dose protocols. Wideochir Inne Tech Maloinwazyjne.

[BIBR-26] Tsukada H., Satou T., Iwashima A., Souma T. (2000). Diagnostic accuracy of CT‐guided automated needle biopsy of lung nodules. AJR Am J Roentgenol.

[BIBR-27] Lee Y.H. (2018). An overview of meta‐analysis for clinicians. Korean J Intern Med.

[BIBR-28] McInnes M.D.F., Moher D., Thombs B.D. (2018). Preferred reporting items for a systematic review and meta‐analysis of diagnostic test accuracy studies: the PRISMA‐DTA statement. JAMA.

[BIBR-29] Whiting P.F., Rutjes A.W., Westwood M.E. (2011). QUADAS‐2: a revised tool for the quality assessment of diagnostic accuracy studies. Ann Intern Med.

[BIBR-30] Anila K.R., Nayak N., Venugopal M., Jayasree K. (2018). Role of rapid on‐site evaluation in CT‐guided fine needle aspiration cytology of lung nodules. J Cytol.

[BIBR-31] Fassina A., Corradin M., Zardo D. (2011). Role and accuracy of rapid on‐site evaluation of CT‐guided fine needle aspiration cytology of lung nodules. Cytopathology.

[BIBR-32] Liu W., Xu C., Li L. (2022). The value of computed tomography‐guided per‐ cutaneous lung biopsy combined with rapid on‐site evaluation in diagnosis of peripheral pulmonary nodules. Technol Cancer Res Treat.

[BIBR-33] Peng T.F., Ren T., Wang H.S. (2020). Diagnostic value of rapid on‐site evaluation for CT‐guided percutaneous fine needle aspiration in the diagnosis of pulmonary occupying lesions. Biomed Res Int.

[BIBR-34] Santambrogio L., Nosotti M., Bellaviti N. (1997). CT‐guided fine needle aspiration cytology of solitary pulmonary nodules: a prospective, randomized study of immediate cytologic evaluation. Chest.

[BIBR-35] Zlevor A.M., Mauch S.C., Knott E.A. (2021). Percutaneous lung biopsy with pleural and parenchymal blood patching: results and complications from 1112 core biopsies. J Vasc Interv Radiol.

[BIBR-36] Yarmus L., Kloot T., Lechtzin N. (2011). A randomized prospective trial of the utility of rapid on‐site evaluation of transbronchial needle aspirate specimens. J Bronchology Interv Pulmonol.

